# Addressing hydrocephalus in Africa: Challenges and way forward

**DOI:** 10.1002/hsr2.1759

**Published:** 2023-12-18

**Authors:** Burhan Kantawala, Maha Khattab, Shaima O. Elawad, Mohamad Assker, Batoul Cherri, Abubakar Nazir, Magda Wojtara, Olivier Uwishema

**Affiliations:** ^1^ Oli Health Magazine Organization, Research and Education Kigali Rwanda; ^2^ Department of Medicine, Faculty of General Medicine Yerevan State Medical University Yerevan Armenia; ^3^ Department of Medicine, Faculty of Medicine Horus University Egypt; ^4^ Department of Medicine, Faculty of Medicine University of Khartoum Khartoum Sudan; ^5^ Department of Medicine, Faculty of Medicine University of Sharjah Sharjah United Arab Emirates; ^6^ Department of Medicine, Faculty of Medicine Lebanese University Beirut Lebanon; ^7^ Department of Medicine King Edward Medical University Lahore Pakistan; ^8^ University of Michigan Medical School Ann Arbor Michigan USA

**Keywords:** Africa, congenital, hydrocephalus, intracranial pressure, traumatic injury

## Abstract

Hydrocephalus occurs when the cerebrospinal fluid (CSF) accumulates in the cerebral ventricles. This is due to either obstruction in the CSF flow, decreasing its absorption by the arachnoid villus to the Dural venous sinuses, or increasing production of the CSF. The most disproportionately and severely affected by the disease consequences are African children. This is because of the high incidence of postinfectious hydrocephalus and spinal dysraphism compared with other world children. The health care system in Africa has access to 488 neurosurgeons which represents less than 1% of the global neurosurgeons, thus pediatric hydrocephalus is considered an emerging public health problem in Africa because of the difficulty of the patient's access to proper care. Numerous studies conducted in Africa have revealed a significant imbalance in the distribution of neurosurgical resources across the continent. Specifically, South Africa and North Africa collectively account for 86% of the total practicing neurosurgeons, indicating a pronounced concentration of these specialized medical professionals in these regions. Having an abundance of case studies regarding hydrocephalus is vital to increase our awareness and understanding. Hydrocephalus should gain more priority by current policymakers as an important health concern. This may be achieved by proper resource allocation to ensure better quality means of diagnosis, intervention, and rehabilitation.

## 1 INTRODUCTION

Hydrocephalus occurs when the cerebrospinal fluid (CSF) accumulates in the cerebral ventricles. This is due to either obstruction in the CSF flow, decreasing its absorption by the arachnoid villus to the Dural venous sinuses, or increasing production of the CSF. Hydrocephalus exhibits various classifications, and in the context of adults, it encompasses four distinct types: obstructive, communicating, hypersecretory, and normal pressure hydrocephalus, whereas in children, there are two classifications: congenital or developmental hydrocephalus.^1^

Hydrocephalus can lead to neurological dysfunctions impacting quality of life. African children are disproportionately affected due to high rates of postinfectious hydrocephalus (PIH) and spinal dysraphism. Limited access to neurosurgical services in African nations accentuates this issue. Thus, this study aims to enhance understanding of hydrocephalus causes, prevalence, etiology, challenges, and treatment across Africa.^2^

## 2 CAUSES AND PREVALENCE OF HYDROCEPHALUS IN AFRICA

**Figure 1:**
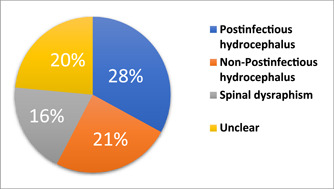
The incidence rate of each category of hydrocephalus in Africa.

In a comprehensive analysis that included studies from 18 African nations, the four categories of causes of hydrocephalus in Africa were identified:

(A). PIH, the most prevalent type of hydrocephalus in Africa, is an acquired condition. Meningitis, ventriculitis, cerebellar abscess‐mastoiditis, severe malaria, basal cyst cryptococcosis, toxoplasmosis infection, and brain and ventricular abscess are among the majority of the conditions that can lead to PIH.
(B). Non‐PIH (NPIH) includes cases of hydrocephalus caused by aqueduct stenosis, Dandy–Walker malformation, tumors, hemorrhages, Chiari malformation (Type 1), and other conditions.
(C). Spinal dysraphism: This group includes hydrocephalus associated with spina bifida, meningocele, myelomeningocele, neural tube defects, (Arnold) Chiari malformation type 2, and spinal dysgraphia.
(D). Unclear: All unclassified and unknown causes of hydrocephalus fall under this category^2^ (Figure 1).

Insufficient information exists regarding the prevalence and incidence of hydrocephalus in Africa. According to a systematic review, Africa has the highest pooled estimated incidence of congenital hydrocephalus [145 per 100,000 births].^3^

**Figure 2:**
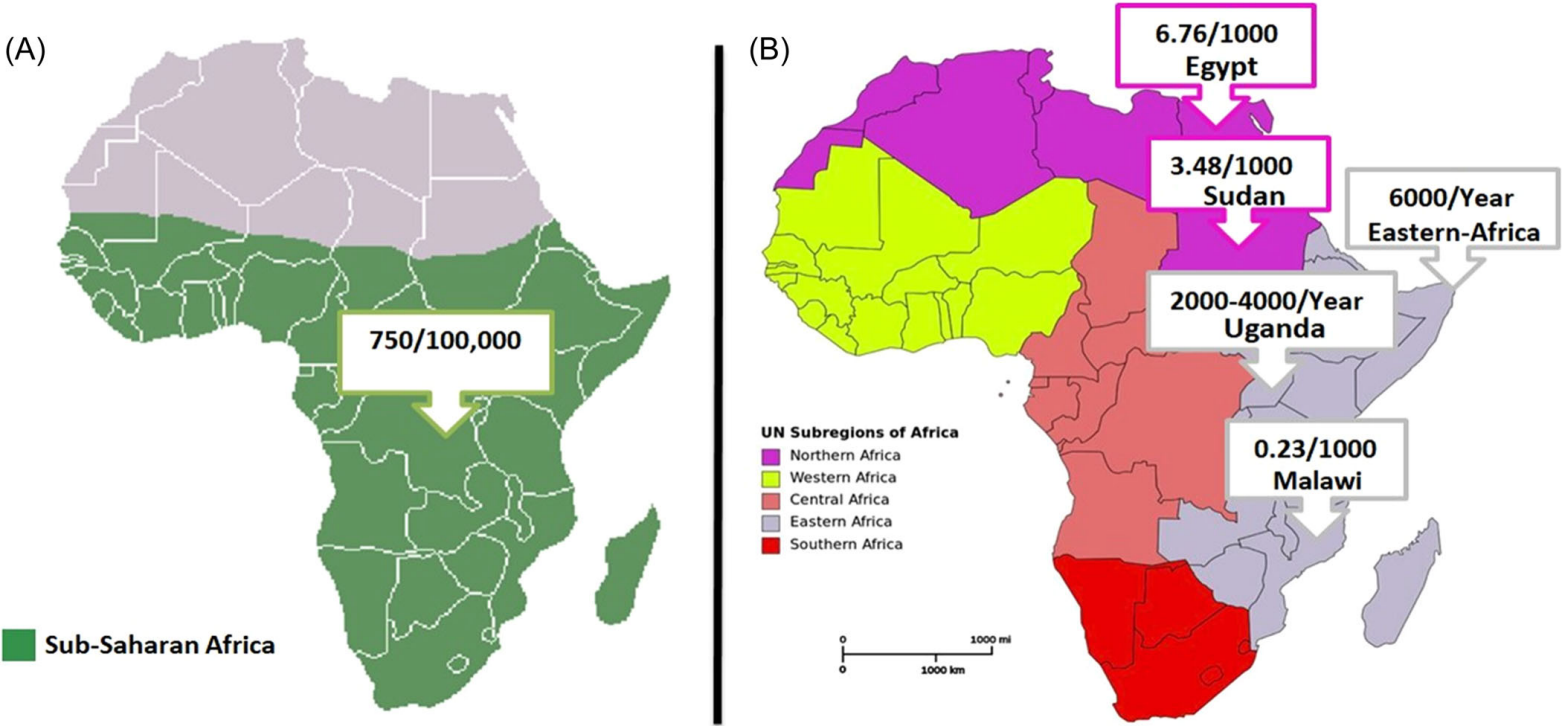
The Incidence of Hydrocephalus in different regions of Africa. (A) Incidence of Hydrocephalus in Sub‐Saharan Africa. (B) The Incidence of hydrocephalus in Eastern Africa and Northern Africa.^3^, ^6^, ^7^, ^8^

In Nigeria, 32% of congenital neurosurgery problems are related to hydrocephalus.^4^ The most common surgical disease burden among young male patients in Kenya is hydrocephalus.^5^ Moreover, 59% of neurosurgical problems in Uganda were hydrocephalus.^6^) The incidence in East Africa is also significant, with more than 6,000 new cases estimated annually^6^ (Figure 2).

## 3 CHALLENGES AND BARRIERS TO TREATMENT

Aside from the high incidence of hydrocephalus in Africa, African countries face significant challenges in treating hydrocephalus patients.^9^, ^10^ Several factors have been identified as being associated with the increased burden of hydrocephalus care. One of the most important factors contributing to the disease's burden is a lack of access to health care facilities and specialists.^9^, ^10^ According to several African studies, the distribution of neurosurgical resources across the continent is unequal, with South Africa and North Africa accounting for 86% of practicing neurosurgeons.^9^ Estimates from Sub‐Saharan African countries, showed that there is one neurosurgeon for every five million people,^11^ compared with one neurosurgeon for every 100,000 people in European countries.^12^

Financial constraints and a lack of insurance coverage can also exacerbate hydrocephalus treatment in African countries. However, financial support for hydrocephalus and other neurosurgical conditions in developing countries, including Africa, is frequently lacking.^9^, ^10^ The cost of transportation are also important considerations when evaluating the economic impact of hydrocephalus.^9^, ^10^ Many patients are forced to travel for hours or even days to reach the nearest primary care facility. They must then be referred to a neurosurgical unit, which often involves several more days of waiting and additional hours of travel time.^10^

Social acceptance of people with visible disabilities remains a problem in several African countries, which can lead to parental delays in seeking healthcare.^10^ Religious beliefs, including the curse concept, are still commonly associated with children suffering from hydrocephalus.^10^

To tackle the challenges associated with hydrocephalus treatment in African countries a multifaceted approach is essential. First, it is imperative to prioritize the enhancement of neurosurgery education across Africa,^13^ and the training of additional pediatric neurosurgeons.^9^ Viable solutions should include enhancing financial support for neurosurgical education, regionalizing educational opportunities, and facilitating the exchange of expertise with high‐capacity centers both within and outside Africa. Moreover, collaborative partnerships between African training centers and their international counterparts will offer substantial benefits for trainees and patients on the continent. The sustainability of these partnerships is more likely when they take into account the specific African context, particularly through collaborations with low‐ and middle‐income countries.^13^

There is an urgent need to alter procurement practices and pricing to make neurosurgery more accessible for families. This could include the use of low‐cost generic replacements for vital drugs, as well as the elimination of trade markups. Furthermore, addressing indirect healthcare costs through transportation vouchers or aiding families with cash transfers for housing can greatly reduce the financial burden associated with healthcare.^14^ It is crucial to build community‐based interventions. Therefore, by including primary healthcare provider education programs, these initiatives empower the frontline medical personnel to identify hydrocephalus symptoms, enable fast referrals to specialists, and facilitate more efficient diagnosis and treatment.^9^

## 4 ENHANCING DIAGNOSIS AND TREATMENT OPTIONS

**Figure 3:**
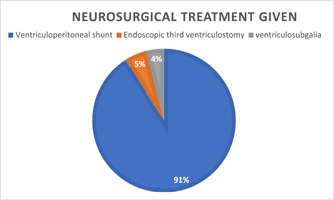
Frequency of surgical treatment for hydrocephalus in Africa.^2^

The health care system in Africa has access to 488 neurosurgeons,^15^ which represents <1% of the global neurosurgeons; thus, pediatric hydrocephalus is considered an emerging public health problem in Africa because of the difficulty of the patient's access to proper care. In 2021, the publication of the Comprehensive Policy Recommendations for the Management of Spina Bifida and Hydrocephalus in Low‐ and Middle‐income Countries document aimed to promote investment in diverse treatment modalities for hydrocephalus. One such efficacious surgical intervention is CSF diversion through the use of shunts or third ventriculostomy, either with or without choroid plexus cauterization.^2^ These interventions serve to mitigate the impact of hydrocephalus and alleviate the associated burden effectively. However, in the future, some types of hydrocephalus could be curable or even prevented by medication instead of surgical treatment, which will be more cost‐efficient for African children due to difficult access to radiology and neurosurgical interventions^2^ (Figure 3).

Early diagnosis of hydrocephalus is crucial to reduce the neurodegenerative residual and psychomotor delay. Unfortunately, most of the hydrocephalus cases are shown late to the outpatient clinics. This is because of the insufficient medical services and the low economic status in African countries. In addition to misdiagnoses of infectious diseases, for instance, bacterial meningitis is one of the most common causes of post‐infectious hydrocephalus.^16^

Irrespective of hydrocephalus etiology, cerebrospinal (CSF) shunts have been widely employed, albeit with a notable malfunction rate: 30% within the first year and 10% annually thereafter. Consequently, Endoscopic Third Ventriculostomy emerges as a favorable choice, especially for infants, despite potential complications. Endoscopic aqueductoplasty stands as an alternative procedure to restore CSF dynamics.^17^

In Sub‐Saharan Africa (SSA), ethical and social complexities surround the treatment of severe hydrocephalus, stemming from inadequate pediatric neurosurgery fellowships, limited medical budgets prioritizing infectious disease treatment such as malaria and tuberculosis. Consequently, insufficient funds hinder early intervention, considering the high mortality rate within 5 years postsurgery. Neuropediatric surgery necessitates sophisticated equipment, including computed tomograpgy scanners, microscopes, and endoscopes, requiring well‐trained medical professionals. Economically, it may not be viable for SSA countries to cater to numerous hydrocephalus patients.^16^

## 5 CASE STUDIES, SUCCESS STORIES, IMPROVING AWARENESS, AND EDUCATION

Restoration of proper CSF flow is the main goal behind most interventions in patients suffering from hydrocephalus. The underlying etiology affects the choice of management greatly. Taenia multiceps is a type of tapeworm responsible for human coenurosis, which in turn causes the sequela of acute hydrocephalus. Formation of multiple cysts in the basal cistern and fourth ventricle contributed to obstructive hydrocephalus in a reported case,^18^ which was not responsive to prolonged antihelminthic therapy. The resolution began upon surgical resection of cysts especially ones in the fourth ventricle accompanied by ventriculoperitoneal (VP) shunt instillation. Multiple VP shunt revisions were made due to repeated obstructions which eventually lead to patient deterioration and death despite initial improvement.

In the presented case study by Labuschagne et al.,^18^ a 5‐year‐old child residing in an area endemic to *Taenia solium* taeniosis/cysticercosis exhibited a month‐long history of vomiting, headaches, developmental regression, and seizures. Initial magnetic resonance imaging (MRI) findings revealed characteristic cysts in CSF spaces, particularly in the basal cisterns, causing acute obstructive hydrocephalus.^18^ These cysts exhibited typical MRI signal characteristics, confirming the diagnosis of neurocysticercosis. The child underwent emergency ventriculo‐peritoneal shunt placement and received empiric treatment with albendazole, praziquantel, and prednisone, initially improving his condition. Despite initial improvement, the child experienced recurrent symptoms, leading to multiple ventriculo‐peritoneal shunt revisions and eventual cachexia. In an attempt to alleviate his intractable symptoms, a minimally invasive transventricular, trans‐aqueductal endoscopic aspiration of the cysts was performed, providing partial relief. Regrettably, despite these interventions, the child's condition continued to deteriorate, ultimately resulting in his demise.^18^

Hydrocephalus may also exist in syndromic presentations, which can complicate its management. In South Africa, presentation of a case of Crouzonodermoskeletal syndrome was diagnosed on the basis of craniofacial features.^19^ The hydrocephalus associated with this condition was managed by shunt operation which was secondary to cranial suture excision as a management for the patient's Craniostenosis, and proper confirmation by molecular investigations to observe FGFR3 mutations was still underway to account for genetic management options.

In this case by Jeftha et al.,^19^ a female infant of European descent, born in Cape Town in 2001, presented with a complex medical history. Antenatal ultrasounds initially indicated concerns about a trilobar skull configuration, raising suspicion of Trisomy 18. However, subsequent chromosomal tests confirmed normal karyotypes. An elective cesarean section was performed at 38 weeks of gestation, leading to the birth of a newborn displaying typical craniofacial characteristics associated with Crouzon syndrome, including mid‐facial hypoplasia and craniosynostosis. Choanal atresia resulted in breathing and feeding difficulties, necessitating a tracheostomy. Management of craniostenosis was achieved through cranial suture excisions. Subsequently, hydrocephalus developed, requiring a shunt operation. By the age of 2 years, the child exhibited additional features of Crouzon syndrome, such as orodental anomalies, midface hypoplasia, and retrognathism. Additionally, acanthosis nigricans was observed, manifesting as dark skin pigmentation in the axillae and groin. Radiographic assessments indicated shortening of tubular bones, especially in the distal phalanges. The family history revealed no consanguinity; although the mother and maternal grandmother displayed mild proptosis, they were otherwise normal in appearance, intellect, and stature.^19^

## 6 CONCLUSION

To conclude, the complexity of hydrocephalus management in Africa requires a comprehensive approach. Having an abundance of case studies regarding hydrocephalus is vital to increase our awareness and understanding. Hydrocephalus should gain more priority by current policymakers as an important health concern. This may be achieved by proper resource allocation to ensure better quality means of diagnosis, intervention, and rehabilitation.^20^, ^21^

Exchanging knowledge, combined with capacity building from experienced healthcare providers globally can help drive the future of hydrocephalus management in Africa to a better place. Further research extending beyond case reports is required to meticulously determine regional variations in both prevalence and incidence, as well as treatment outcomes. The future of addressing hydrocephalus in Africa is promising as long as it is accompanied by collaborative efforts aiming to provide a better quality of life for persons affected by hydrocephalus.

This may be achieved by accumulating a wealth of case studies on hydrocephalus, as it provides insights into diverse scenarios within the continent. Also, international partnerships, where experienced healthcare providers globally work with African counterparts, can encompass training programs and telemedicine consultations, which thoroughly examines regional variations in hydrocephalus prevalence and incidence, ensuring quality diagnosis, intervention, and rehabilitation.

## AUTHOR CONTRIBUTIONS

**Burhan Kantawala**: Writing—original draft; writing—review and editing. **Maha Khattab**: Writing—original draft; writing—review and editing. **Shaima O. Elawad**: Writing—original draft; writing—review and editing. **Mohamad Assker**: Writing—original draft; writing—review and editing. **Batoul Cherri**: Writing—original draft; writing—review and editing. **Abubakar Nazir**: Supervision; writing—original draft. **Magda Wojtara**: Writing—original draft; writing—review and editing. **Olivier Uwishema**: Supervision; validation.

## CONFLICT OF INTEREST STATEMENT

The authors declare no conflict of interest.

## ETHICS STATEMENT

Not applicable.

## TRANSPARENCY STATEMENT

The lead author Abubakar Nazir affirms that this manuscript is an honest, accurate, and transparent account of the study being reported; that no important aspects of the study have been omitted; and that any discrepancies from the study as planned (and, if relevant, registered) have been explained.

## Data Availability

Not applicable.
